# Increase of cells expressing PD-L1 in bovine leukemia virus infection and enhancement of anti-viral immune responses in vitro via PD-L1 blockade

**DOI:** 10.1186/1297-9716-42-103

**Published:** 2011-09-26

**Authors:** Ryoyo Ikebuchi, Satoru Konnai, Tatsuya Shirai, Yuji Sunden, Shiro Murata, Misao Onuma, Kazuhiko Ohashi

**Affiliations:** 1 Department of Disease Control, Graduate School of Veterinary Medicine, Hokkaido University, Sapporo, 060-0818, Japan; 2 Department of Veterinary Clinical Sciences, Graduate School of Veterinary Medicine, Hokkaido University, Sapporo, 060-0818, Japan

## Abstract

The inhibitory receptor programmed death-1 (PD-1) and its ligand, programmed death-ligand 1 (PD-L1) are involved in immune evasion mechanisms for several pathogens causing chronic infections. Blockade of the PD-1/PD-L1 pathway restores anti-virus immune responses, with concomitant reduction in viral load. In a previous report, we showed that, in bovine leukemia virus (BLV) infection, the expression of bovine PD-1 is closely associated with disease progression. However, the functions of bovine PD-L1 are still unknown. To investigate the role of PD-L1 in BLV infection, we identified the bovine PD-L1 gene, and examined PD-L1 expression in BLV-infected cattle in comparison with uninfected cattle. The deduced amino acid sequence of bovine PD-L1 shows high homology to the human and mouse PD-L1. The proportion of PD-L1 positive cells, especially among B cells, was upregulated in cattle with the late stage of the disease compared to cattle at the aleukemic infection stage or uninfected cattle. The proportion of PD-L1 positive cells correlated positively with prediction markers for the progression of the disease such as leukocyte number, virus load and virus titer whilst on the contrary, it inversely correlated with the degree of interferon-gamma expression. Blockade of the PD-1/PD-L1 pathway in vitro by PD-L1-specific antibody upregulated the production of interleukin-2 and interferon-gamma, and correspondingly, downregulated the BLV provirus load and the proportion of BLV-gp51 expressing cells. These data suggest that PD-L1 induces immunoinhibition in disease progressed cattle during chronic BLV infection. Therefore, PD-L1 would be a potential target for developing immunotherapies against BLV infection.

## Introduction

The immune response to bovine leukemia virus (BLV) in cattle is an important factor to determine the outcome of BLV infection. BLV is a B-cell tropic virus that is genetically closely related to human T-cell leukemia virus-1 (HTLV-1). The majority of BLV-infected cattle are clinically inapparent, and are referred to as asymptomatic or aleukemic (AL). A small fraction of the latently infected individuals develop the disease characterized by persistent lymphocytosis (PL) and B cell lymphoma. During BLV-infection especially at the PL and lymphoma stage, T-cell dysfunction including, impaired cell proliferation and cytokine production characterized by the down-regulation of Th1 cytokines, accelerates the disease progression through mechanisms yet to be elucidated [1-3].

An immunoinhibitory receptor programmed death-1 (PD-1) and its ligand, programmed death-ligand 1 (PD-L1) are involved in immune dysfunction in several chronic infections and cancers [4,5]. In particular, recent in vitro and in vivo studies have shown the importance of the PD-1/PD-L1 pathway in retroviral infections, such as human immunodeficiency virus (HIV), HTLV-1 and simian immunodeficiency virus (SIV). PD-1 and PD-L1, whose expression is upregulated on CD4^+ ^and CD8^+ ^T cells specific for HIV [6,7] and HTLV-1 [8], negatively regulate T-cell activation through the inhibition of a T cell receptor signal. Moreover, blocking of the PD-1/PD-L1 pathway by antibodies specific to PD-1 or PD-L1 has been shown to restore T cell function during HIV and HTLV infection in vitro [6,8,9]. Interestingly, in the SIV model for potential immunotherapy, the viral load was significantly reduced by the inoculation of anti PD-1 antibody in vivo [10,11]. These findings indicated that high expression of PD-1 and PD-L1 in retrovirus infection leads to T cell dysfunction, suggesting that the reinvigoration of immune dysfunction has a potential for application in clinical immunotherapy against these chronic infections.

To determine the contribution of the PD-1/PD-L1 pathway to immune dysfunction caused by several domesticated animal diseases such as BLV, we have previously cloned bovine PD-1 and shown that the expression profiles of PD-1 in CD4^+ ^and CD8^+ ^T cells are closely associated with BLV-induced lymphoma [12]. However, the dynamics and functions of PD-L1 in disease progression during BLV infection remain unknown. In this study, in an attempt to determine whether the PD-1/PD-L1 system promotes the BLV-induced immunosuppression, we cloned, sequenced and characterized the cDNA encoding bovine PD-L1, and subsequently measured the expression levels of bovine PD-L1 in BLV-infected cattle at different disease stages. We also investigated the effects of blockade of PD-1/PD-L1 by anti-PD-L1 antibody on BLV infection.

## Materials and methods

### Cell preparation, subset isolation and depletion

Bovine peripheral blood mononuclear cells (PBMC) were purified from heparinized venous blood of healthy Holstein-Friesian and Japanese Black breed, maintained at the Graduate School of Veterinary Medicine, Hokkaido University, by density gradient centrifugation on Percoll (Amersham Pharmacia Biotech, Piscataway, NJ, USA). CD4^+ ^T cell, CD8^+ ^T cell, CD5^+ ^cell and CD14^+ ^monocyte populations were isolated from PBMC using the BD IMag™ Cell Separation System (BD Biosciences, Franklin Lakes, NJ, USA) and the following antibodies: CACT138A (mouse anti-bovine CD4: VMRD, WA, USA), IL-A51 (mouse anti-bovine CD8: a gift from International Livestock Research Institute), CACT105A (mouse anti-bovine CD5: VMRD) and CAM36A (mouse anti-bovine CD14: VMRD), respectively. T cell subsets (CD4^+^, CD8^+^, CD3^+^) were depleted from PBMC using the following antibodies: CACT138A, IL-A51 and MM1A (mouse anti-bovine CD3: VMRD). The purity of each cell population was confirmed by EPICS XL flowcytometry system (Beckman Coulter, Inc., Fullerton, CA, USA) with the EPICS EXPO32 ADC software (Beckman Coulter). Highly purified cells (> 90%) were used for the analysis of the PD-L1 expression and T cell-depleted PBMC (< 5%) were used for the PD-L1 blockade assay.

### Cloning of full length cDNA encoding bovine PD-L1 and sequence analysis

To induce PD-L1 expression, PBMC were incubated at 37°C for 4 h with 5% CO2 in the presence of 5 μg/mL concanavalinA (ConA) (Sigma-Aldrich, St. Louis, MO, USA). Total RNA was extracted from cultivated PBMC using the Trizol reagent (Invitrogen, Carlsbad, CA, USA) according to the manufacturer's instructions, and residual DNA was removed from the RNA samples by treatment with Deoxyribonuclease I (Invitorogen). cDNA was synthesized from the RNA samples using Moloney murine leukemia virus reverse transcriptase (Takara, Shiga, Japan) according to the manufacturer's instructions. 3'- and 5'- rapid amplification of cDNA ends (RACE) were performed using the 3'-/5'- RACE System for Rapid Amplification of cDNA Ends (Invitrogen) with PD-L1 gene-specific primers, 5'-ACG TGT CAG GCT GAG GGT TAC CCT GAA GC-3' and 5'-GTC ACA TTT TTC TAC ATC-3'. After obtaining the 5'- and 3'-end sequences, we designed new primers, 5'-ATG AGG ATA TAT AGT GTC TT-3', and 5'-TTA CGT CTC CTC AAA CTG T-3', and performed PCR to clone the full length bovine PD-L1 cDNA. The resulting amplification products were cloned into the pGEM-T Easy vector (Promega, Madison, WI, USA), and sequenced using CEQ2000 DNA Analysis System (Beckman Coulter). The established sequences were aligned, and an unrooted neighbor-joining tree was constructed with the Mega 4 software [13].

### Expression analysis of bovine PD-L1 mRNA by quantitative real-time PCR

To investigate the expression levels of *PD-L1 *mRNA, RNA samples were extracted from purified CD4^+^, CD8^+^, CD5^+^, CD14^+ ^cells and PBMC incubated for 18 h in the presence of either anti-bovine CD3 antibody (0.2 μg/mL, MM1A, VMRD) or ConA (5 μg/mL). cDNA was synthesized from the RNA samples as described above. Quantitative RT real-time PCR was performed using the LightCycler 480 system II (Roche Diagnostics, Mannheim, Germany) according to the manufacturer's instructions. The cDNA template was mixed with 10 μL of SYBR Premix DimerEraser (Takara) and 0.6 μL each of primers (10 pmol/μL) in a total volume of 20 μL. Primers used were 5'-GGG GGT TTA CTG TTG CTT GA-3' and 5'-GCC ACCT CAG GAC TTG GTG AT-3' for bovine PD-L1, and 5'-CGC ACC ACT GGC ATT GTC AT-3' and 5'-TCC AAG GCG ACG TAG CAG AG.-3' for β-actin. The cycling conditions consisted of initial template denaturing at 95°C for 30 s, followed by the amplification of template for 45 cycles of 95°C 5 s, 60°C 30 s and 72°C 30 s. A final melting curve analysis was performed from 65 to 95°C at the rate of 0.1°C/s (continuous acquisition), with the final cooling to 40°C for 10 s. Each amplification procedure was done in triplicate, and the results of *PD-L1 *mRNA expression are presented as a ratio obtained by dividing the concentration of the *PD-L1 *mRNA by that of the β-*actin *mRNA.

### BLV-infection diagnosis

Blood samples from Japanese black, Holstein-Friesian and First filial of Japanese black/Holstein-Friesian were investigated. All of the cattle from which the blood samples were obtained had been diagnosed with BLV-infection at the Veterinary Teaching Hospital, Graduate School of Veterinary Medicine, Hokkaido University, between 2008 and 2010. Genomic DNA was extracted from whole blood using the Wizard™ Genomic DNA Purification kit (Promega). The concentration of purified genomic DNA was measured at OD of 260 nm, and the DNA samples were stored at 4°C until use. BLV infection was tested by nested-PCR to amplify the BLV long terminal region (LTR) using primer pairs BLV-LTR1 5'-TGT ATG AAA GAT CAT GCC GAC-3' and BLV-LTR533 5'-AAT TGT TTG CCG GTC TCT-3' for the initial PCR, and BLV-LTR256 5'-GAG CTC TCT TGC TCC CGA GAC-3' and BLV-LTR453 5'-GAA ACA AAC GCG GGT GCA AGC CAG-3' for the second PCR. The conditions for both of the PCR were incubations at 94°C for 5 min, followed by amplification of template for 35 cycles of 94°C 30 s, 55°C 30 s and 72°C 30 s with the final extension at 72°C for 7 min. The provirus load was further confirmed by real-time PCR using a LightCycler 480 system II, with SYBR Premix DimerEraser and primers, BLV-LTR256 and BLV-LTR453 for BLV, and 5'-ACA CAA CTG TGT TCA CTA GC-3' and 5'-CAA CTT CAT CCA CGT TCA CC-3' for bovine β-globin, as described previously [14]. 100% provirus load corresponded to each PBMC having one copy of BLV provirus. Furthermore, the virus titers were quantified based on the number of syncytia formed by isolated PBMC co-cultured with a mouse sarcoma virus-transformed (cc81) feline cell line. The syncytium formation assay was conducted according to the procedure described previously [2]. The 1 × 10^5 ^cc81 cell line was grown with 5 × 10^5 ^PBMC from BLV-infected animals for 72 h in a 24-well plate (Corning, NY, USA) with RPMI medium (Invitrogen) containing 10% heat-inactivated fetal calf serum (Invitrogen), 2 mmol L-glutamine, 100 U/mL penicillin and 100 μg/mL streptomycin. The confluent cells were then fixed in methanol for 30 min and stained with 10% Giemsa solution (Merck, Darmstadt, Germany) for 30 min. All samples were tested in triplicate and the data are presented as the mean numbers of syncytia.

To investigate the degree of immunosuppression in cattle, *interferon *(*IFN*)*-γ *mRNA was quantified by quantitative RT real-time PCR as described above. The primers used were 5'-ATA ACC AGG TCA TTC AAA GG-3' and 5'-ATT CTG ACT TCT CTT CCG CT-3'. Cattle with lymphoma were diagnosed clinically, and confirmed by microscopic and histological examinations. Other BLV-positive animals were classified as AL or PL based on the amount of leukocytes and the percentage of B-cells in their whole blood as described previously [15].

### Flow cytometry analysis

To analyze the cells expressing PD-L1, single- and dual-color flow cytometric analysis was performed using the following antibodies; CACT105A (mouse anti-bovine CD5), BIG73A (mouse anti-bovine IgM, VMRD) and H-130 (rabbit anti-human PD-L1; Santa Cruz Biotechnology, Santa Cruz, CA, USA) as previously described [16]. Purified PBMC (1 × 10^7 ^cells/mL) were incubated with the optimal concentration of each antibody for 40 min at 4°C. Then, the cells were washed with PBS containing EDTA (0.5 mg/mL) and stained with either FITC-conjugated goat anti-rabbit IgG (Santa Cruz) or PE-conjugated goat anti-mouse IgG (Beckman Coulter). Rabbit IgG isotype control (Beckman Coulter) and normal mouse serum were used as isotype controls. Fluorescence of the cells was measured on an EPICS XL flowcytometry system (Beckman Coulter), and the data were analyzed using the EPICS EXPO32 ADC software (Beckman Coulter).

### PD-L1 blockade assay

For PD-L1 blockade, PBMC and T cell-depleted PBMC from BLV-infected cattle (4 × 10^6 ^cells/well in 24-well plates) were cultured at 37°C for 18 h (for cytokine mRNA expression analysis) or 72 h (for the evaluation of inhibitory effects on virus propagation) with ConA (5 μg/mL) in the presence of either 10 μg/mL rabbit anti-human PD-L1 antibody or rabbit IgG isotype control. To quantify anti-viral factors, *interleukin *(*IL*)*-2 *and *IFN-γ *mRNA expressions were analyzed in bovine PBMC cultivated for 12~18 h by real-time PCR using primers, 5'-TTT TAC GTG CCC AA GGT TAA-3' and 5'-CGT TTA CTG TTG CAT CAT CA-3' for IL-2, and 5'-ATA ACC AGG TCA TTC AAA GG-3' and 5'-ATT CTG ACT TCT CTT CCG CT-3' for IFN-γ. RNA extraction from the cells, cDNA synthesis and real-time quantitative RT-PCR were conducted as described previously [2]. To examine the influence of the PD-L1 blockade on virus propagation, 72 h-cultivated bovine PBMC were harvested and total DNA was extracted from the cells. Provirus load was measured using real-time PCR as described above. And after 48 h cultivation, BLV-gp51^+ ^cells were analyzed by flow cytometry using the BLV1 (mouse anti-BLV envelope protein gp51, VMRD). The data are indicated as relative ratio to control treated with only ConA.

### Statistics

Spearman rank-correlation, one-way ANOVA with Tukey's post test and Wilcoxon matched pairs test were performed using GraphPad Prism version5.0. *P*-values of less than 0.05 were considered to be statistically significant.

## Results

### Sequence analysis of bovine PD-L1

**Figure 1:**
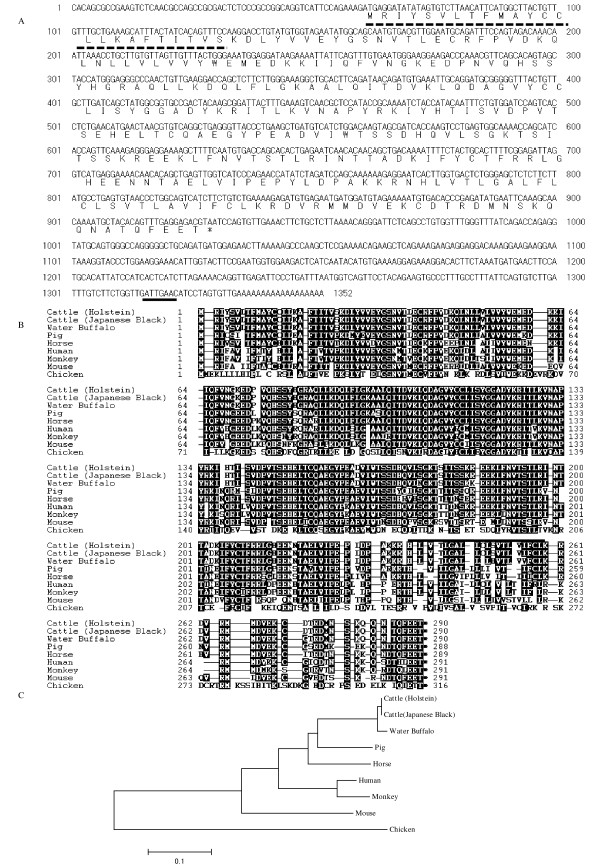
**Cloning of bovine PD-L1**. A: Nucleotide and deduced amino acid sequences of bovine PD-L1 cDNA. Bovine PD-L1 cDNA has an open reading frame extending from positions 61 to 930 and encodes for a 290 aa polypeptide. The putative signal peptide and polyadenylation signal are underlined with dotted and solid lines, respectively. B: Alignment of deduced amino acid sequences of vertebrate PD-L1. Bold line indicates the transmembrane domain (238-259) of bovine PD-L1. C: Phylogenetic tree constructed from nucleotide sequences of vertebrate PD-L1 by the Mega 4 software. The bootstrap values supporting a particular cluster are shown within the node. Numbers indicate the bootstrap percentage (100 replicates). The scale indicates the divergence time. Clusters were identified based on their Order.

To clone the bovine PD-L1 gene, total RNA isolated from bovine PBMC was used to synthesize total cDNA from which the full-length sequence of bovine PD-L1 cDNA was generated by 5'- and 3'- RACE. The full-length bovine PD-L1 cDNA sequence and its putative amino acid sequence obtained from the Holstein breed is shown in Figure 1a [GenBank: AB510902]. The complete nucleotide sequence was 1 352 bp in length, and contained 60 nucleotides upstream of the initial ATG codon and 422 nucleotides downstream of the stop-codon. The polyadenylation site was located at 13 bases upstream of the poly A tail. The PD-L1 cDNA sequence derived from Japanese Black breed cattle was found to be identical to that obtained from Holstein breed cattle (Table 1). In comparison to other artiodactyls species, the predicted amino acid sequence of bovine PD-L1 was found to be most similar (99.0%) to that of water buffalo. The bovine PD-L1 had amino acid identities of 92.0%, 89.3%, 85.2%, 83.4%, 80.7% and 52.1% to the pig, horse, human, monkey, mouse and chicken PD-L1, respectively (Table 1). Sequence analysis of bovine PD-L1 shows that the amino acid residues 1 to 24 represent a putative signal peptide, and that the residues 238 to 259 represent the transmembrane domain (Figure 1b). Phylogenetic analysis revealed that vertebrate PD-L1 was divided into two groups, mammalian (Primate, Rodentia, Carnivora and Artiodactyla) and avian groups, with bovine PD-L1 clustering in the artiodactyl species group (Figure 1c).

**Table 1:** The nucleic and amino acid sequence similarities (%) of PD-L1 among animal species

Species (GenBank accession number)	Cattle (Holstein)	Cattle (Japanese Black)	Water Buffalo	Pig	Horse	Human	Monkey	Mouse	Chicken
Cattle (Holstein)	-	100	98.3	88	87.5	83.3	82	73.5	56.3
Cattle (Japanese Black)	100	-	98.3	88	87.5	83.3	82	73.5	56.3
Water Buffalo (FJ827147)	99	99	-	87.8	87.4	82.6	81.7	73.8	55.6
Pig (NM 001025221)	92	92	92.4	-	88.9	84.7	83.5	74.5	55.2
Horse (XM 001492842)	89.3	89.3	89.6	91.3	-	86.9	85.6	75.5	55.9
Human (AK314567)	85.2	85.2	85.2	86.9	86.6	-	96	76.5	55
Monkey (EF444816)	83.4	83.4	84.1	85.2	85.5	96.2	-	75.5	55
Mouse (AF317088)	80.7	80.7	81.4	82.4	81.4	82.8	82.1	-	55.5
Chicken (XM 424811)	52.1	52.1	52.1	54.6	55.2	54.3	55.2	53	-

Upper section shows percent homologies in nucleic acid level, and lower section shows those in deduced amino acid level. GenBank accession numbers are described under species name.

### Comparative analysis of PD-L1 expression in various bovine PBMC-derived cell types

**Figure 2:**
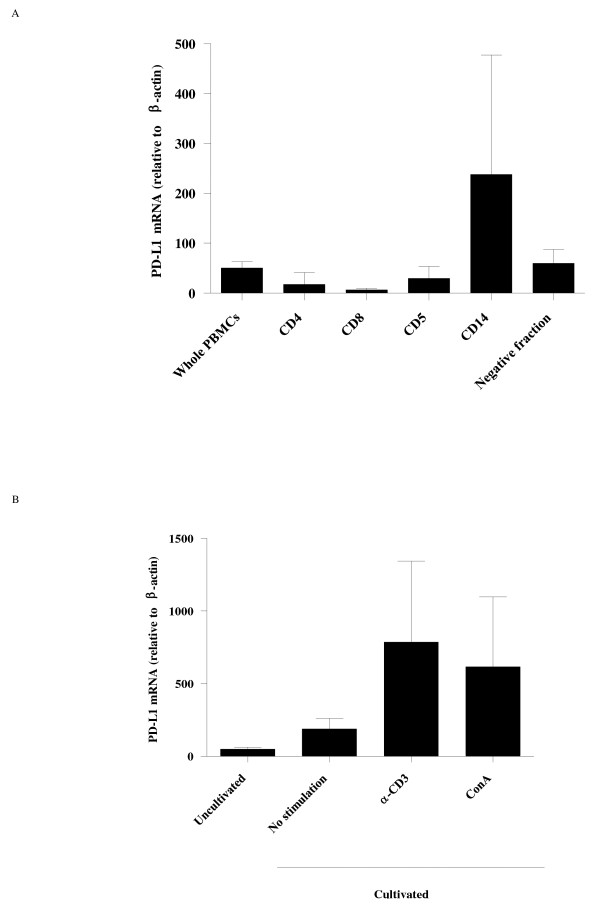
**Analysis of bovine *PD-L1 *mRNA expression by real-time PCR**. A: *PD-L1 *mRNA expressions were determined in total PBMC from three healthy Holstein cattle, subpopulations of CD4^+^, CD8^+^, CD5^+^, CD14^+ ^cells and negative fraction for these subpopulations. B: *PD-L1 *mRNA expression in PBMC incubated with or without either anti-bovine CD3 antibody (αCD3) or ConA. The level of the *PD-L1 *mRNA expression was shown as the ratios obtained by dividing concentrations of the PCR products from *PD-L1 *mRNA by those from β-*actin *mRNA. Error bars represent the SD of the means among the three cows.

In order to investigate the expression levels of bovine *PD-L1 *mRNA, we used real-time PCR to quantify the expression of bovine *PD-L1 *mRNA in PBMC. Firstly, we evaluated the expression levels of bovine *PD-L1 *mRNA in several cell types among PBMC (mean ± SD: 50.67 ± 12.63) from healthy cattle (Figure 2a). Among the PBMC cell subsets, the expression of *PD-L1 *mRNA in CD14^+ ^cells (238.0 ± 239.5), which are representative of monocytes, was higher than that in CD5^+ ^cells (the T cells and B-1 cells fraction) (29.83 ± 24.06), CD4^+ ^T cells (17.65 ± 23.67) and CD8^+ ^T cells (6.793 ± 3.122). The *PD-L1 *mRNA expression was also observed in the negative fraction (60.37 ± 27.45), which was considered to contain natural killer cells and conventional B cells among others. To determine the effect of T cell stimulation on the expression of bovine *PD-L1 *mRNA, PBMC from healthy animals were cultured in the presence of anti-CD3 monoclonal antibody. As shown in Figure 2b, the addition of this antibody enhanced the expression of bovine *PD-L1 *mRNA in PBMC (785.7 ± 557.4) to a magnitude similar to that induced by ConA (616.7 ± 481.3), a lectin which induces T cell proliferation by its specific interaction with the T cell receptor complex. There were no notable differences in the expression levels of bovine *PD-L1 *mRNA before and after cell cultivation without stimulation (187.7 ± 73.28).

### PD-L1 expression in BLV-infected cattle at different disease stages

**Figure 3:**
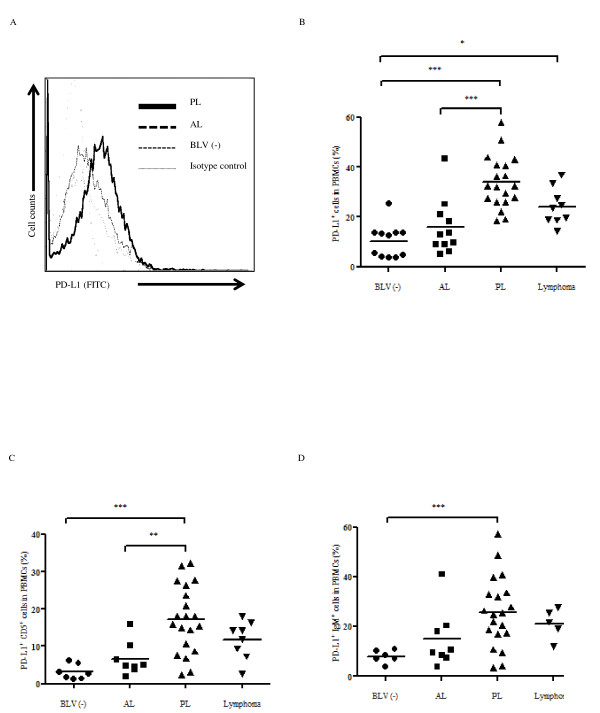
**Flow cytometric analysis of bovine PD-L1 expression in BLV-infected cattle**. A: Typical cases of different expression levels of PD-L1 in BLV-infected cattle at different disease stages determined by flow cytometric analysis. Bold lines indicate the up-regulation of the PD-L1 expression in the PL animal. B, C, D: Flow cytometric analysis of the PD-L1 expression on PBMC (B), CD5^+ ^(C), and IgM^+ ^(D) cells during the BLV-induced disease progression. PBMC from BLV-negative (BLV (-): *n *= 11 in B, 5 in C and D) and BLV-infected cattle with AL (*n *= 11 in B, 8 in C and D), PL (*n *= 19) and lymphoma (*n *= 9 in B, 8 in C, 5 in D) were analyzed. Individual dots indicate percentages of PD-L1^+ ^cells in PBMC from BLV-uninfected (circle), AL (square), PL (upward triangle) and lymphoma (downward triangle). Each line indicates the mean percentages in each group. Statistical comparisons were made using the one-way ANOVA with Tukey's post test. Differences between groups were considered statistically significant at probability values of *P *< 0.05 (* *P *< 0.05; ** *P *< 0.01; *** *P *< 0.001).

To evaluate PD-L1 expression in BLV-infected animals, we examined the percentages of PD-L1^+ ^cells in PBMC freshly isolated from BLV-infected cattle at different disease stages. Typical cases of different expression levels of PD-L1 in BLV-infected cattle at different disease stages determined by flow cytometric analysis are shown in Figure 3a. The mean percentage of PD-L1^+ ^cells in PBMC isolated from PL cattle (33.89 ± 10.55) was significantly higher than that of uninfected cattle (10.32 ± 6.801, *p *< 0.001) and AL cattle (15.81 ± 11.05, *p *< 0.001) (Figure 3b). Similarly, the mean percentage of PD-L1^+ ^cells was significantly increased in cattle with lymphoma (24.17 ± 7.404) compared to BLV-negative cattle (*p *< 0.05). No difference in the mean percentage of PD-L1^+ ^cells was observed between BLV-negative and AL cattle, as well as between PL and cattle with lymphoma. In the case of retrovirus infections such as HIV and HTLV-1, the up-regulation of the PD-L1 expression has been observed in target cells for virus infection or infected cells [8,17]. BLV is characterized as a B-cell tropic virus and induces aberrant B cell (especially CD5^+ ^or IgM^+ ^B cell) proliferation during disease progression. As an attempt to evaluate the expression of PD-L1 on B cells, we measured the proportion of the cells expressing PD-L1 among IgM^+ ^cells and CD5^+ ^cells by flow cytometry. As expected, the number of IgM^+ ^cells and CD5^+ ^cells were increased during disease progression (data not shown). As shown in Figures 3c and 3d, the percentages of PD-L1^+ ^and CD5^+ ^or IgM^+ ^cells in PBMC were clearly increased in cattle at the PL stage (17.22 ± 9.004 and 25.61 ± 14.13) compared to BLV-negative (3.154 ± 1.989, *p *< 0.001 and 7.935 ± 2.613, *p *< 0.001) and AL cattle (6.623 ± 4.479, *p *< 0.01 and 14.86 ± 11.91). The proportions of PD-L1^+ ^and CD5^+ ^or IgM^+ ^cells isolated from cattle with lymphoma (11.77 ± 5.122 and 21.23 ± 6.091) were elevated compared to those of AL and BLV-negative cattle, although the differences were not statistically significant.

### Correlation between the number of leukocytes, provirus loads or virus titer and PD-L1 expression

**Figure 4:**
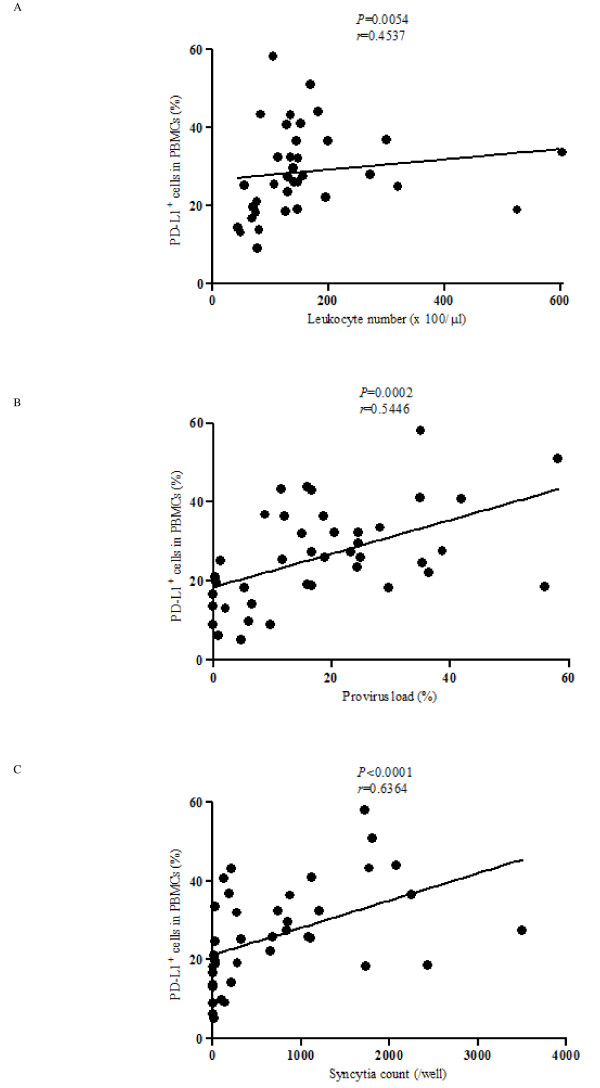
**Correlation between markers of disease progression and PD-L1 expression in BLV-infected cattle**. Positive correlation between the leukocyte number (A: *n *= 36; AL: 8 PL: 20 EBL: 8), provirus load (B: *n *= 41; AL: 12 PL: 20 EBL: 9) or virus titer (C: *n *= 39; AL: 12 PL: 19 EBL: 8) and the percentages of PD-L1^+ ^cells in PBMC corresponding to Figure 3b. The provirus load and virus titer were determined using quantitative real-time PCR and syncytium formation assay, respectively. Correlation statistics were analyzed using the Spearman correlation.

To determine if the increased proportion of PD-L1-positive cells among PBMC correlated to the changes in the number of leukocytes, provirus loads and virus titer in BLV-infected cattle, we performed statistical analysis based on Pearson correlation coefficients (Figure 4). As expected, a significant positive correlation was detected between the levels of PD-L1^+ ^cells and the number of leukocytes (Figure 4a: *p *< 0.01). Notably, the levels of PD-L1^+ ^cells correlated with provirus loads (Figure 4b: *p *< 0.001) and virus titer (Figure 4c: *p *< 0.0001) in BLV-infected cattle. These results suggest that an increase of PD-L1^+ ^cells could influence the expansion of BLV-infected cells during the progression of the disease.

### Correlation between IFN-γ expression levels and PD-L1 expression

**Figure 5:**
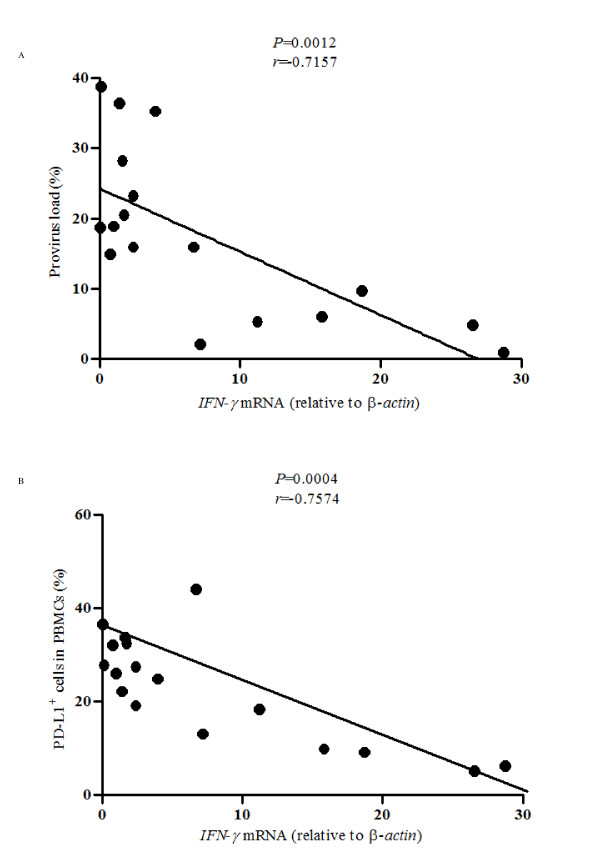
**Correlation between PD-L1 and IFN-γ expression in BLV-infected cattle**. Inverse correlation between IFN-γ expression and provirus load (A), and IFN- γ expression and percentages of PD-L1^+ ^cells in PBMC (B) (*n *= 17; AL: 6 PL: 8 EBL: 3). Quantification of the mRNA expression for IFN- γ in isolated PBMC from BLV-infected cattle was done by real-time PCR analysis. The level of *IFN- γ *mRNA expression is shown as the ratios obtained by dividing the concentrations of the PCR products from *IFN- γ *mRNA by those from β-*actin *mRNA. Correlation statistics were analyzed using the Spearman correlation.

It has been known that IFN-γ, a key cytokine for virus clearance, is downregulated during BLV infection [2]. This phenomenon is characterized as immune suppression that facilitates disease progression during BLV infection through an unknown mechanism. Thus, we investigated the correlation between the percentages of PD-L1^+ ^cells and IFN- γ expression in PBMC from BLV infected animals. As observed in the previous study [2], increased IFN-γ levels correlated significantly with reduced provirus load in BLV-infected animals (Figure 5a: *p *< 0.01). Interestingly, lower proportions of circulating PD-L1^+ ^cells correlated strongly with increased IFN-γ levels (Figure 5b: *p *< 0.001). These results prompted the notion that decreased levels of IFN-γ during virus proliferation and progression of BLV-induced disease could be due to the increase of PD-L1^+ ^cells.

### Effect of the PD-L1 blockade on cytokine expression and virus propagation

**Figure 6:**
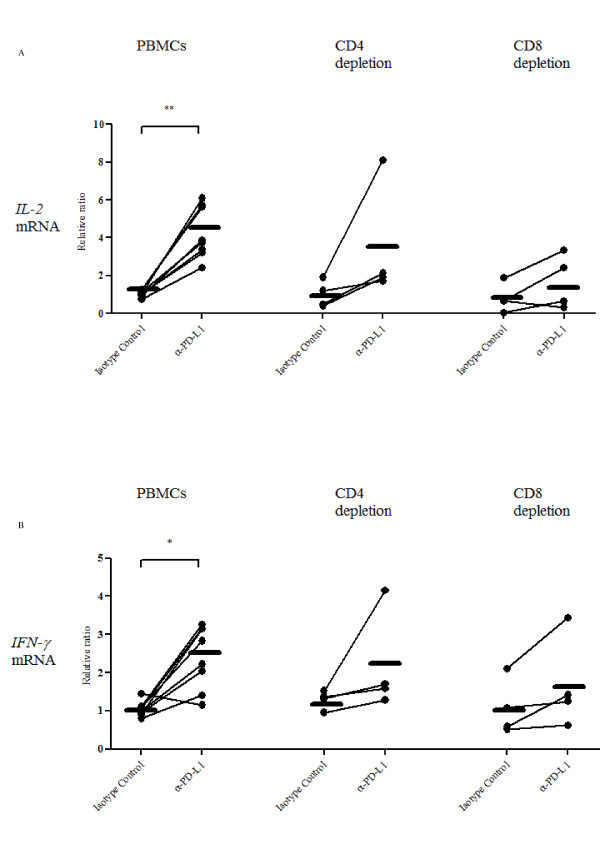
**Blockade of the PD-1/PD-L1 pathway increases anti-virus immune responses**. Upregulations of IL-2 (A) and IFN-γ (B) expressions by the blockade of the PD-1/PD-L1 pathway using anti-PD-L1 antibody (α-PD-L1). The cytokine expression in cultivated PBMC (*n *= 8; PL: 5 EBL: 3) and CD4^+ ^or CD8^+ ^cell-depleted PBMC (*n *= 4; PL: 4) were determined using quantitative real-time PCR. The results are indicated as relative change to control (no antibody treated). Each line indicates the mean expression level in each group. Statistical comparisons between Isotype Control and α-PD-L1 were made using the Wilcoxon matched pairs test and those between PBMC and T cell-depleted PBMC were made using the one-way ANOVA with the Tukey's post test. Differences between groups were considered statistically significant at probability values of *P *< 0.05 (* *P *< 0.05; ** *P *< 0.01).

**Figure 7:**
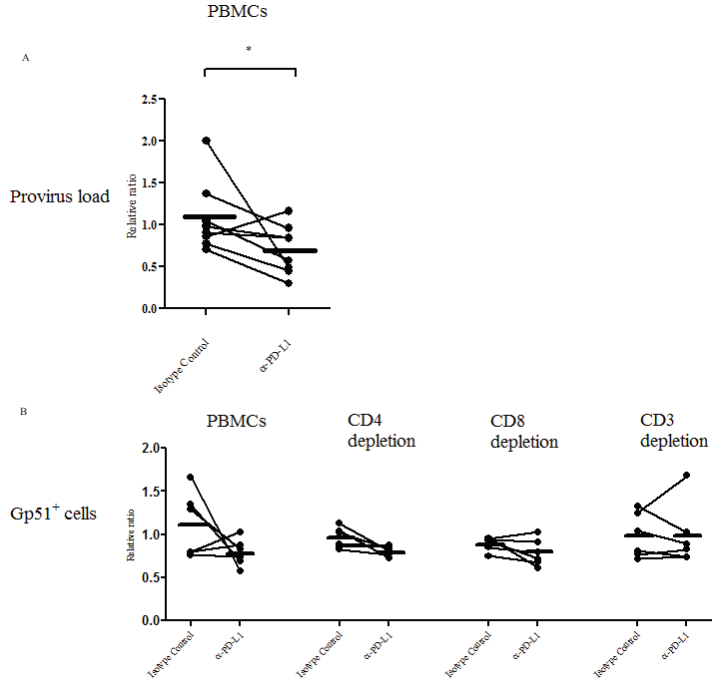
**Blockade of the PD-1/PD-L1 pathway decreases virus load and proportion of BLV-gp51**^**+ **^**cells**. Downregulations of provirus load in PBMC (A: *n *= 8; PL: 5 EBL: 3) and proportions of BLV-gp51^+ ^cells in PBMC and T cell-depleted PBMC (B: *n *= 6; PL: 6) by the blockade of the PD-1/PD-L1 pathway using anti-PD-L1 antibody (α-PD-L1). The provirus loads in cultivated PBMC were determined using quantitative real-time PCR. The proportions of BLV-gp51^+ ^cells were determined using flow cytometry. The results are indicated as relative change to control (no antibody treated). Each line indicates the mean expression level in each group. Statistical comparisons between Isotype Control and α-PD-L1 were made using the Wilcoxon matched pairs test and those between PBMC and T cell-depleted PBMC were made using the one-way ANOVA with the Tukey's post test. Differences between groups were considered statistically significant at probability values of *P *< 0.05 (* *P *< 0.05).

To test the effect of PD-1/PD-L1 blockade on immune cell function, PBMC from BLV-infected cattle were cultivated in the presence of ConA together with either an anti-PD-L1 or an isotype control antibody. The expression of anti-viral cytokine (IL-2 and IFN-γ) mRNA in the cultured PBMC were analyzed by quantitative real-time PCR. The expression of both IL-2 and IFN-γ was found to be augmented in cells treated with the anti-PD-L1 antibody compared to those treated with the control antibody for 18 h (Figure 6). *IL-2 *mRNA was increased by 4.258 fold (Figure 6a: *p *< 0.01), while *IFN-γ *mRNA was increased by 2.401 fold (Figure 6b: *p *< 0.05). In contrast, there was no notable change in cytokine expression in PBMC treated with the isotype control antibody. To confirm that the PD-L1 blockade can enhance T cell dependent functions, CD4^+ ^or CD8^+ ^cell-depleted PBMC were cultivated in the presence of either an anti-PD-L1 or an isotype control antibody. A limited number of cattle were tested, the restorative effect of IL-2 (Figure 6a) and IFN-γ (Figure 6b) expression were lower in the T cell-depleted PBMC than those of whole PBMC, although there is no significant difference between them. These findings raise the possibility that the PD-1/PD-L1 blockade could inhibit BLV propagation. Thus, we examined the provirus load and proportion of the BLV-gp51^+ ^cells in the presence of either an anti-PD-L1 or an isotype control antibody. As expected, the provirus load was strongly decreased (except in one case) by the addition of anti-PD-L1 antibody (Figure 7a: 0.69 ± 0.29, *p *< 0.05). BLV-gp51^+^cells were notably eliminated by the addition with anti-PD-L1 antibody (0.79 ± 0.16), although some individual differences were observed (Figure 7b). The anti-viral effects were downregulated by T cell-depletions, such as CD4^+^, CD8^+ ^and CD3^+ ^cells, although no significant difference was observed, so the results need to be confirmed using a larger sample size of cattle (Figure 7b).

## Discussion

In domestic animals including cattle, there are still many intractable diseases with poor-prognosis because of lack of effective treatment and vaccination. This may be attributed to the lack of a better understanding of immunological mechanisms leading to immune evasion. Recent studies have indicated the involvement of a major inhibitory network of the PD-1/PD-L1 pathways in immune dysfunction in several human diseases. However, there are only a few reports available on the role of the PD-1/PD-L1 pathways in domestic animals. As an example, porcine PD-1 and PD-L1 have been cloned and shown to have functions [18,19]. In the present study, the genes for bovine PD-L1 in two breeds of cattle were cloned and sequenced, and together with bovine PD-1 reported in our previous study [5], they were compared to the sequences from various mammalian species. We also demonstrated the correlation between BLV infection and PD-L1 expression, and confirmed that the blockade of PD-L1 by anti-PD-L1 antibody led to upregulation of cytokine production that resulted in enhancement of anti-virus immunity and ultimately reduced virus load and infected cells in vitro. A deeper understanding of the PD-1/PD-L1 pathway in domestic animals will facilitate the elucidation of events leading to immune dysfunction during the progression of incurable diseases.

In our studies, PD-L1 expression on PBMC, especially B cells that proliferated abnormally, and PD-1 expression on CD4^+ ^and CD8^+ ^T cells [5] were found to be upregulated in cattle with advanced disease, consistent with previous reports showing the upregulation of PD-1 and PD-L1 expression in retrovirus infections. In HIV infection, the expression of PD-L1 has been found to be upregulated on myeloid DC [17] as well as on CD4^+ ^and CD8^+ ^effector T cells [7]. Moreover, HIV-specific CD4^+ ^and CD8^+ ^T cells have been shown to express high levels of PD-1 that correlated with HIV-specific T-cell dysfunction with impaired proliferative responses to virus-derived antigens [6,9,20]. In HTLV infection, the upregulation of PD-L1 on CD4^+ ^and CD25^+ ^T cells, as well as the upregulation of PD-1 on CD8^+ ^T cells (especially HTLV-1-specific T cells) together with anti-virus T cell dysfunctions have been reported [8,21]. BLV, which is related to HTLV, causes chronic infections of B cells that may lead to leukemia and lymphoma in cattle through mechanisms of disease progression yet to be elucidated. Nevertheless, there has been evidence of dysfunction of cellular immunity that has been associated with progression of the disease [2]. Thus, our findings underscore earlier reports suggesting that the PD-1/PD-L1 pathway in retrovirus infection is associated with T cell dysfunction during disease progression, and that PD-L1^+ ^B cells proliferate abnormally in BLV-infected cattle. However, in the present study, we could not compare the expression of PD-L1 on BLV-infected cells between cattle at different disease stages because BLV-gp51 was not found to be expressed on freshly isolated PBMC from infected cattle, even at the late stage of the disease. Nevertheless, after a short period of culturing the cells, BLV-gp51 could be expressed on B cells, but the co-founding complication was that the downregulation of PD-L1 expression appeared to be inconsistent among the various cattle tested. It was thus difficult to determine whether the expression of PD-L1 was specifically upregulated on BLV-infected B cells compared to BLV-negative B cells, despite its clear effect on the provirus load and virus titer. Further detailed analyses will be needed to confirm this.

The mechanism of PD-L1 upregulation during BLV-induced disease progression remains unknown. In HIV models, several hypotheses on what elevates PD-1 and PD-L1 expressions, such as cytokine microenvironment [22], virus-derived proteins [23], influx of microbial products [24] and chronic antigen presentation [25], have been proposed. These hypotheses might contribute to understanding the mechanism of PD-L1 upregulation during BLV infection, considering that changes in cytokine microenvironment [1,2,26,27] and aberrant modulation of host molecules by a BLV transcription factor, Tax, have been previously reported in BLV-infected animals [28]. Nevertheless, further elucidation of the mechanism for the elevation of PD-L1 expression is warranted to fully understand the cell signaling pathways involved in the modulation of host immune responses.

In mouse models, the reinvigoration of exhausted anti-viral immune responses by the blockade of the PD-1/PD-L1 pathway is well established. Blockade of the PD-1/PD-L1 pathway by anti-PD-L1 antibody, presumably by blocking the inhibitory signal of PD-1, results in the enhancement of the proliferative capacity of T cells specific to lymphocytic choriomeningitis virus (LCMV), hepatitis C virus and hepatitis B virus [29-32]. Similar observations have been made for HIV and HTLV infections by using antibodies to block the PD-1/PD-L1 interaction in in vitro cell cultures [6,8,20]. In the present study, we have shown that blockade of PD-1/PD-L1 by anti-PD-L1 antibody upregulated the expression of anti-virus cytokines (IFN-γ and IL-2) in cultured PBMC that had been isolated from immune-suppressed BLV-infected cattle. It is noteworthy that the blockade of the PD-1/PD-L1 pathway restored anti-viral immune functions and reduced BLV provirus and BLV-gp51^+ ^cells in vitro. The results obtained in this study were consistent with those of LCMV, HIV and HTLV.

As an alternative method to confirm that the PD-L1 blockade can enhance T cell functions, CD4^+ ^or CD8^+ ^cell depleted PBMC were cultivated in the presence of anti-PD-L1 antibody. A limited number of cattle were tested, yet as expected, the restorative effect of IL-2 and IFN-γ expression were lower in the T cell depleted PBMC than those of PBMC. In addition, BLV-gp51^+^cells were notably eliminated by the addition of anti-PD-L1 antibody, and the anti-viral effects were downregulated by T cell depletions. The likely cause of the inhibition of BLV proliferation during the blockade of the PD-1/PD-L1 pathway in vitro could be due to the enhancement of proliferation of CTL and Th1 cells that have anti-virus specific cellular functions. However, our observations could not point to anti-BLV specific immune cells. Bovine or sheep T cell epitope peptides of BLV gp51 were identified in several works [33,34], so the analysis of the PD-1/PD-L1 blockade stimulated by the epitope peptide or the peptide mix of other BLV protein helped to clarify whether the BLV specific immune reaction is upregulated or not. Furthermore, by using real-time RT-PCR, presumed secreted anti-viral factors, granzyme B and perforin, were analyzed in PBMC cultivated in the presence of anti-PD-L1 antibody. However, no significant changes in the expression profiles of these anti-viral factors were observed in the PBMC containing cells expressing high levels of *IFN-γ *and *IL-2 *mRNA (data not shown). Further elucidation of the detailed mechanisms concerning the effect of the PD-1/PD-L1 blockade on BLV-induced disease progression is required.

It remains to be determined whether bovine PD-L1 on B cells has any function similar to mouse or human PD-L1 on T and B cells whose functions are to send the immunoinhibitory signals into the cells. The intracellular domain of mouse, human and bovine PD-L1 is only about 30 aa in length and there is no known functional domain, suggesting that PD-L1 may function only as a ligand. However, there have been some conflicting reports on the intracellular signaling functions of mouse and human PD-L1. Stimulation of PD-L1 on Epstein-Barr virus-transformed B cells has been reported to induce apoptosis of the B cells [35], while PD-L1 on cancer cells has been shown to act as an anti-apoptotic receptor [36]. PD-L1 on mouse and human T cells has been demonstrated to interact with B7-1 (CD80), resulting in the generation of the inhibitory signal in the T cells [37,38]. Although no consensus was reached about the PD-L1 function as a receptor in our study, the treatment with anti-PD-L1 antibody induced the reduction in provirus load, suggesting the presence of a reverse-signaling mechanism through PD-L1.

In conclusion, we present here an aberrant expression of PD-L1 on B-cells in BLV-infected cattle, with the elevated proportion of PD-L1^+ ^cells correlating with immune evasion during the course of the disease. Consistent with previous reports, the blockade of the PD-1/PD-L1 pathway enhanced T-cell function and resulted in the inhibition of BLV proliferation. Thus, PD-1-induced T-cell dysfunction through its binding to the PD-L1 on B-cells might contribute to disease progression. The treatment of SIV-infected macaques and LCMV-infected mice with either PD-L1 or PD-1 antibody have restored multiple functions in formerly exhausted T cells and resulted in virus clearance in vivo [10,11,29]. Thus, blocking the PD-1/PD-L1 pathway has a potential clinical application in enhancing host antimicrobial immunity to treat chronic infections. Studies are underway to evaluate the possible clinical application of the PD-1/PD-L1 blockade as a novel strategy to control bovine diseases using the BLV infection model.

## List of Abbreviations

AL: aleukemic; BLV: bovine leukemia virus; ConA: concanavalinA; CTL: Cytotoxic T-lymphocyte; HIV: human immunodeficiency virus; HTLV-1: human T-cell leukemia virus-1; IFN: interferon; IL: interleukin; LCMV: lymphocytic choriomeningitis virus; LTR: long terminal region; PBMCs: peripheral blood mononuclear cells; PD-1: programmed death-1; PD-L1: programmed death-ligand 1; PL: persistent lymphocytosis; RACE: rapid amplification of cDNA ends; SIV: simian immunodeficiency virus

## Competing interests

The authors declare that they have no competing interests.

## Authors' contributions

RI carried out all of the studies contained in this manuscript, analyzed data and drafted the manuscript. SK participated in the experimental design, analyzed data, and helped to draft the manuscript. TS participated in some experiments and sample collection. YS participated in the experimental design and reviewed the manuscript. SM helped with experimental design and data interpretation. MO helped in the analysis of the data, overall guidance of the studies. KO supervised the study and reviewed the manuscript. All authors read and approved the final manuscript.
